# Ethically robust reproductive genetic carrier screening needs to measure outcomes that matter to patients

**DOI:** 10.1038/s41431-022-01109-7

**Published:** 2022-05-18

**Authors:** Lisa Dive, Ainsley J. Newson

**Affiliations:** 1 The University of Sydney, Faculty of Medicine and Health, Sydney School of Public Health, Sydney Health Ethics, Sydney, NSW 2006 Australia; 2 Present Address: Graduate School of Health, University of Technology Sydney, NSW 2007 Broadway, Australia

**Keywords:** Ethics, Population genetics

Identifying appropriate outcomes for programs such as reproductive genetic carrier screening (RGCS) is important. Knowing which outcomes should be measured or evaluated is vital for assessing program success. Richardson et al. in this issue [1] review qualitative data on patient-reported outcomes in RGCS and provide valuable insights to inform the development of a core outcome set for RGCS.

In addition to being important for evaluating program success, outcome sets should also reflect the underlying goals of interventions like RGCS. In this commentary we reflect on the way that outcomes for RGCS might shift as it becomes more like a screening intervention offered at population scale. In considering this shift, we contend both that outcome sets must be strongly tied to program goals, and that program goals should, in turn, reflect the ethical underpinning of such a program.

As yet, there is no consensus as to the goals of RGCS, and debate on this continues [2]. As Richardson et al. note, carrier testing began as a clinical intervention, available to those with a known higher chance (based on family history or ethnicity) of having children with an inherited condition. The goals of RGCS in this context often included avoiding the birth of a child with the specific genetic condition. However targeted carrier testing is now evolving towards population-level screening, offered independent of pre-existing risk. Next-generation sequencing has also enabled many conditions to be screened simultaneously.

Clinicians and those with lived experience of serious genetic conditions tend to support the goal of reducing the suffering associated with such conditions [3, 4]. However the goal of prevention in an expanded and widely available screening program is ethically contestable [5]. When RGCS is offered at population scale, it arguably implies that the conditions included in screening are worth taking steps to avoid. Without careful program design, this implication can reinforce or even exacerbate societal attitudes that place a lesser value on the lives of people who live with disability and difference. Having prevention of the birth of children with a genetic condition as a primary goal of RGCS is therefore ethically problematic.

A compatible – yet more justifiable – goal for RGCS at population scale is to offer RGCS as a way of supporting reproductive decision-making by providing information that might be relevant. Such an approach seeks to avoid implicit judgements either about whether to participate in screening, or which choices to make following a RGCS result. It also reflects that RGCS does not exist solely to enable prospective parents to avoid the birth of a child with a particular condition, as the information can also be useful for managing pregnancy care and early life interventions. The goal is to make information available via RGCS for those couples who would find it valuable for purposes of all kinds of reproductive decision-making.

Outcome sets for RGCS follow directly from identified program goals. Given the debate about acceptable goals for RGCS, outcomes should also necessarily reflect the ethical commitments made in determining such goals. It has been argued that ethically acceptable RGCS must incorporate plural goals that emphasise reproductive autonomy but also pluralistic commitments to public health values, such as solidarity and responding to health inequities [5]. So program outcomes follow (logically) from the goals of RGCS, and these goals need ethical justification.

Given the debate around the acceptability of different goals for RGCS, the patient-identified outcomes highlighted in Richardson et al. are highly relevant. A value-pluralistic approach to RGCS allows for multiple compatible goals including improving the health of mothers and babies, and reducing inequity in access to health interventions. It is generally agreed that fostering reproductive autonomy should be prominent among the goals of RGCS [6]. A key outcome for RGCS should therefore be to provide participants with valuable information that enables informed decision-making. As such, patient perspectives on what they want from RGCS should be reflected in outcome sets.

Of the additional patient-identified outcomes identified in Richardson et al., the domain of “perceived utility of RGCS” seems particularly important, since this reflects the value (for participants) of information obtained from RGCS. Utility of genetic tests can be considered in terms of *clinical utility* – the ability to lead to improved health outcomes – or *personal utility*. Personal utility can be understood as the capacity for a genetic test to produce outcomes that have value to the patient, even if that value is not ‘clinical’ in nature [7]. Genetic tests that produce personal utility might have less ability to influence clinical care, but can help people by increasing their knowledge, enabling them to communicate more effectively with family, and feel that they are coping better [8].

Richardson et al. identify two components of personal utility from the qualitative literature. The first relates to “a sense of confidence and empowerment related to reproductive decisions” (p. 15). The other is more practical: in order for RGCS to produce utility, results must be provided in a timely way so that they can be incorporated into decision-making. While reproductive confidence and empowerment are less prominent in the literature (and require more conceptual bioethics exploration), they are likely to help ensure people feel “in charge” of their reproductive choices, and make considered decisions that align with their goals and values. An analysis of these related concepts is beyond the scope of this commentary, but it seems evident that perceived (personal) utility as an outcome of RGCS is important to a goal of promoting reproductive autonomy.

Future research should continue to attend to the underlying ethical commitments of outcome sets for RGCS, as reflected in their goals. A value pluralistic approach to RGCS emphasises reproductive autonomy as among its goals. Measuring patient-identified outcomes such as those identified by Richardson et al. provides a helpful first step to evaluating the success of ethically robust RGCS programs.

## References

[1] Richardson EJ, McEwen A, Newton-John T, Crook A, Jacobs C Incorporating Patient Perspectives in the Development of a Core Outcome Set for Reproductive Genetic Carrier Screening: A Sequential Systematic Review. Eur J Hum Genet. 2022; 10.1038/s41431-022-01090-1&x2019. 10.1038/s41431-022-01090-1 PMC9259674 35347269

[2] Cornel MC, Rigter T, Jansen ME, Henneman L Neonatal and carrier screening for rare diseases: how innovation challenges screening criteria worldwide. J Community Genet. 2021;12:257–265. 10.1007/s12687-020-00488-y PMC8141077 33074550

[3] Ong R, Howting D, Rea A, Christian H, Charman P, Molster C (2018). Measuring the impact of genetic knowledge on intentions and attitudes of the community towards expanded preconception carrier screening. J Med Genet.

[4] Boardman FK, Young PJ, Griffiths FE (2017). Population screening for spinal muscular atrophy: A mixed methods study of the views of affected families. Am J Med Genet A..

[5] Dive L, Newson AJ (2021). Ethics of reproductive genetic carrier screening: from the clinic to the population. Public Health Ethics.

[6] Henneman L, Borry P, Chokoshvili D, Cornel MC, van El CG, Forzano F (2016). Responsible implementation of expanded carrier screening. Eur J Hum Genet.

[7] Bunnik EM, Janssens AC, Schermer MH (2015). Personal utility in genomic testing: is there such a thing?. J Med Ethics.

[8] Kohler JN, Turbitt E, Biesecker BB (2017). Personal utility in genomic testing: a systematic literature review. Eur J Hum Genet.

